# Glycolytic regulatory enzyme PFKFB3 as a prognostic and tumor microenvironment biomarker in human cancers

**DOI:** 10.18632/aging.204758

**Published:** 2023-05-30

**Authors:** Qingen Da, Lei Huang, Can Huang, Zee Chen, Zhitong Jiang, Fang Huang, Tao Shen, Lu Sun, Zilong Yan, Xiaoqiang Ye, Jing Yi, Yu Huang, JingJing Da, Mingming Ren, Jikui Liu, Tao Wang, Zhen Han, Kunfu Ouyang

**Affiliations:** 1 Department of Cardiovascular Surgery, Peking University Shenzhen Hospital, Shenzhen, Guangdong, China; 2 Department of Hepatobiliary Surgery, Peking University Shenzhen Hospital, Shenzhen, Guangdong, China; 3 Renal Division, Department of Medicine, Guizhou Provincial People’s Hospital, Guiyang, Guangdong, China

**Keywords:** PFKFB3, pan-cancer, TME, immune infiltration, risk prognostic model, kidney renal papillary cell carcinoma

## Abstract

The 6-phosphofructo-2-kinase/fructose-2,6-bisphosphatase 3 (PFK-2/FBPase-2, PFKFB3) is a glycolysis regulatory enzyme and plays a key role in oncogenesis of several cancers. However, the systematic study of crosstalk between PFKFB3 and Tumor microenvironment (TME) in pan-cancer has less been examined. In this study, we conducted a comprehensive analysis of the relationship between *PFKFB3* expression, patient prognostic, Tumor mutational burden (TMB), Microsatellite instability (MSI), DNA mismatch repair (MMR), and especially TME, including immune infiltration, immune regulator, and immune checkpoint, across 33 types of tumors using datasets of The Cancer Genome Atlas (TCGA) and Gene Expression Omnibus (GEO). We found that *PFKFB3* expression was significantly correlated with patient prognostic and TME factors in various tumors. Moreover, we confirmed that PFKFB3 was an independent prognostic factor for kidney renal papillary cell carcinoma (KIRP), and established a risk prognostic model based on the expression of *PFKFB3* as a clinical risk factor, which has a good predictive ability. Our study indicated that PFKFB3 is a potent regulatory factor for TME and has the potential to be a valuable prognostic biomarker in human tumor therapy.

## INTRODUCTION

Glycolysis is an essential enzymatic process in cell metabolism. The substrates produced from glycolysis are required in many metabolic pathways, such as the tricarboxylic acid cycle, pentose phosphate pathway, and nucleotide, amino acid, and lipid synthesis pathways. Cancer cell metabolism is commonly reprogrammed to the glycolytic pathway to address the need for increased glucose uptake and production of lactate. This metabolic switch occurs even when the tumor cells have mitochondria and are in sufficient oxygen conditions for normal oxidative phosphorylation, suggesting that glycolysis plays an important role in tumorigenesis [1–4].

The 6-phosphofructo-2-kinase/fructose-2,6-bisphosphatase (PFK-2/FBPase-2, PFKFB) protein is a major regulator of glycolysis and functions as a bifunctional enzyme that reversibly regulates fructose 2,6-bisphosphate synthesis and degradation [5]. Four isozymes of PFK-2 have been identified: PFKFB1, PFKFB2, PFKFB3, and PFKFB4. PFKFB3 is widely involved in multiple biological processes, such as angiogenesis, DNA damage repair, autophagy, cell cycle, and response to hypoxia [6–9]. The tumor microenvironment (TME) is a key regulatory factor in tumors and contributes to the initiation, progression, and metastasis of tumors [10]. Some studies have demonstrated the aberrant expression of *PFKFB3* in cancer tissues and its role in tumorigenesis [11]. However, the systematic study of crosstalk between PFKFB3 and TME in pan-cancer has less been examined.

In this study, we conducted a comprehensive assessment of the relationship between *PFKFB3* expression, patient prognosis, and especially TME in cancers based on the TCGA database. To study the role of PFKFB3 in tumors, we studied the mRNA and protein expression level, phosphorylation modification, genetic alteration, Tumour mutational burden (TMB), Microsatellite instability (MSI), DNA mismatch repair (MMR), immune infiltration, clinical outcome, the characteristic of expression in a single cell, and function of enrichment of PFKFB3 were used to investigate the potential roles in tumor development.

## RESULTS

### The characteristic of *PFKFB3* expression in pan-cancer

**Figure 1:**
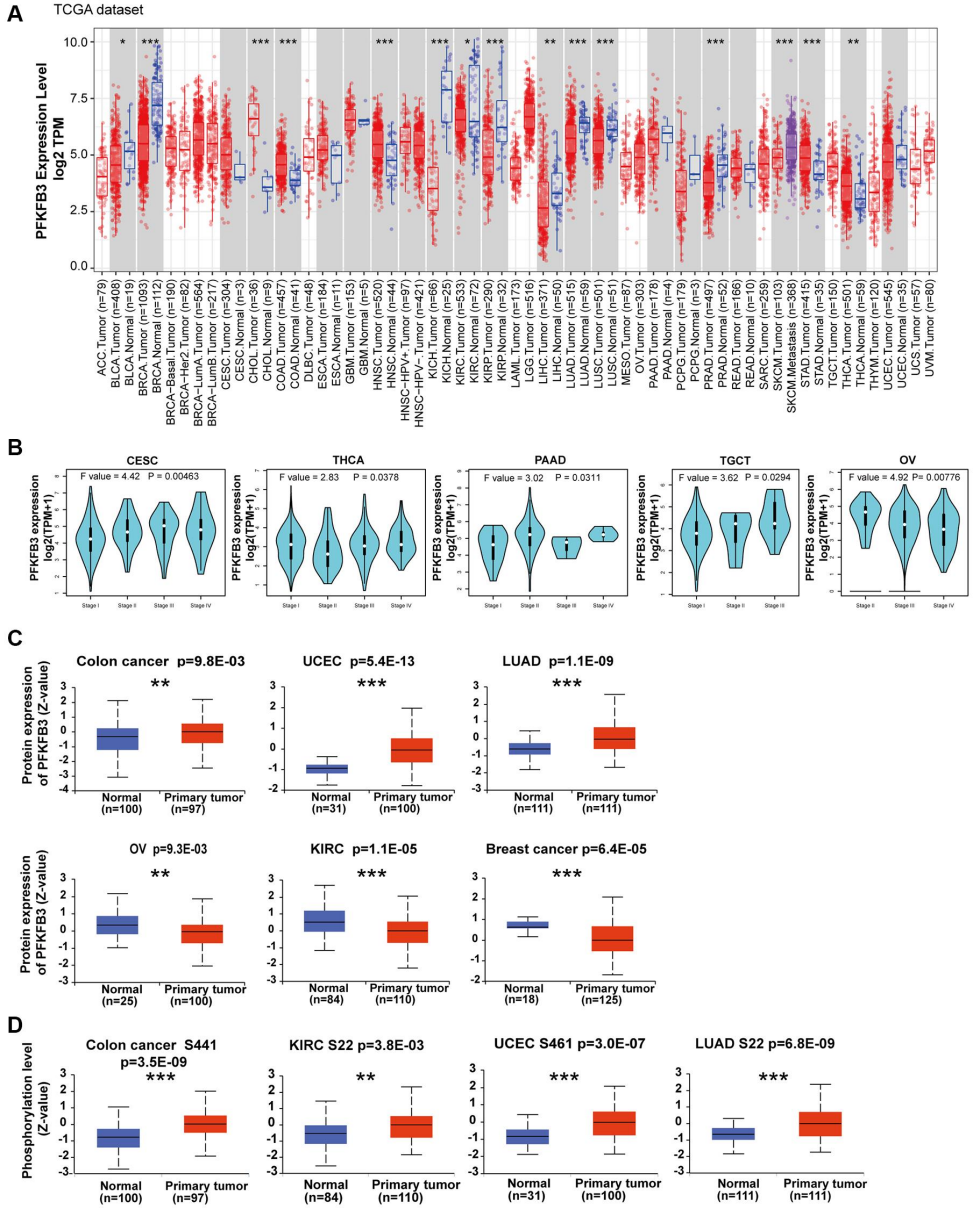
**Expression level and phosphorylation of PFKFB3 in pan-cancer.** (**A**) The expression status of the *PFKFB3* gene in different cancers was analyzed via TIMER2. ^*^P < 0.05; ^**^P < 0.01; ^***^P < 0.001. (**B**) The expression of the *PFKFB3* gene was studied according to the pathological stage (stage I–IV) of the different TCGA cancers, including CESC, THCA, PAAD, TGCT, and OV. (**C**) We analyzed the expression level of PFKFB3 protein in tumor and non-tumor tissues of colon cancer, UCEC, LUAD, ovarian cancer, KIRC, and breast cancer by the CPTAC dataset. ^**^P < 0.01, ^***^P < 0.001. (**D**) We analyzed the phosphorylation level of PFKFB3 (S22, S461, and S441 sites) between primary tumor tissue and non-tumor tissues via the UALCAN. ^**^P < 0.01, ^***^P < 0.001.

We first analyzed the mRNA expression level of PFK-2 family genes across various cancer types in TCGA and GTEx datasets using the GEPIA database. Compared with other PFK-2 family members, the mRNA expression level of *PFKFB3* was much higher than *PFKFB1, PFKFB2,* and *PFKFB4* (Supplementary Figure 1). The mRNA expression level of *PFKFB3* was markedly elevated in tumor tissues of colon adenocarcinoma (COAD), cholangiocarcinoma (CHOL), head and neck squamous cell carcinoma (HNSC), stomach adenocarcinoma (STAD), and thyroid carcinoma (THCA) compared with the respective non-tumor tissues (Figure 1A). However, *PFKFB3* mRNA level was lower in tumor tissues of breast invasive carcinoma (BRCA), bladder urothelial carcinoma (BLCA), kidney renal clear cell carcinoma (KIRC), kidney chromophobe (KICH), kidney renal papillary cell carcinoma (KIRP), liver hepatocellular carcinoma (LIHC), lung squamous cell carcinoma (LUSC), lung adenocarcinoma (LUAD), prostate adenocarcinoma (PRAD), lymphoid neoplasm diffuse large B-cell lymphoma (DLBC), and thymoma (THYM) compared with corresponding non-tumor tissues (Figure 1A and Supplementary Figure 2). Furthermore, strong correlations between *PFKFB3* expression and pathological stage were observed in endocervical adenocarcinoma (CESC), THCA, pancreatic adenocarcinoma (PAAD), testicular germ cell tumors (TGCT), and Ovarian serous cystadenocarcinoma (OV, all *P* < 0.05, Figure 1B and Supplementary Figure 3).

PFKFB3 protein level was analyzed by the CPTAC dataset, and the results showed that the protein level of PFKFB3 in colon cancer (*p* = 9.8E-03), uterine corpus endometrial carcinoma (UCEC, *p* = 5.4E-13), and LUAD (*p* = 1.1E-09) was much higher than in non-tumor tissues, while lower expression of PFKFB3 was observed in OV (*p* = 9.3E-03), KIRC (*p* = 1.1E-05), and breast cancer (*p* = 6.4E-05, Figure 1C).

We have used the UALCAN database to investigate the promoter methylation level of *PFKFB3* in human pan-cancer. We found the promoter methylation level of *PFKFB3* was significantly decreased in TGCT, UCEC, BLCA, LUSC, PRAD, HNSC, THCA, LIHC, and LUAD tissues compared to normal tissues according to the UALCAN database (Supplementary Figure 4A). The methylation level of *PFKFB3* in KIRC, BRCA, COAD, SARC, and KIRP was greatly increased compared to normal tissues (Supplementary Figure 4B).

Combining UALCAN with the CPTAC dataset, we analyzed PFKFB3 phosphorylation levels in five types of tumors (breast cancer, colon cancer, KIRC, LUAD, and UCEC). Phosphorylation of S22 on PFKFB3 was significantly elevated in KIRC (*P* < 0.01) and LUAD (*P* < 0.001) (Figure 1D). Higher phosphorylation at S461 was observed in UCEC (*P* < 0.001), but not in breast cancer. Followed by a significantly increased phosphorylation level of the S441 was found in colon cancer (*P* < 0.001, Figure 1D).

### The survival prognosis value of PFKFB3 in pan-cancer

**Figure 2:**
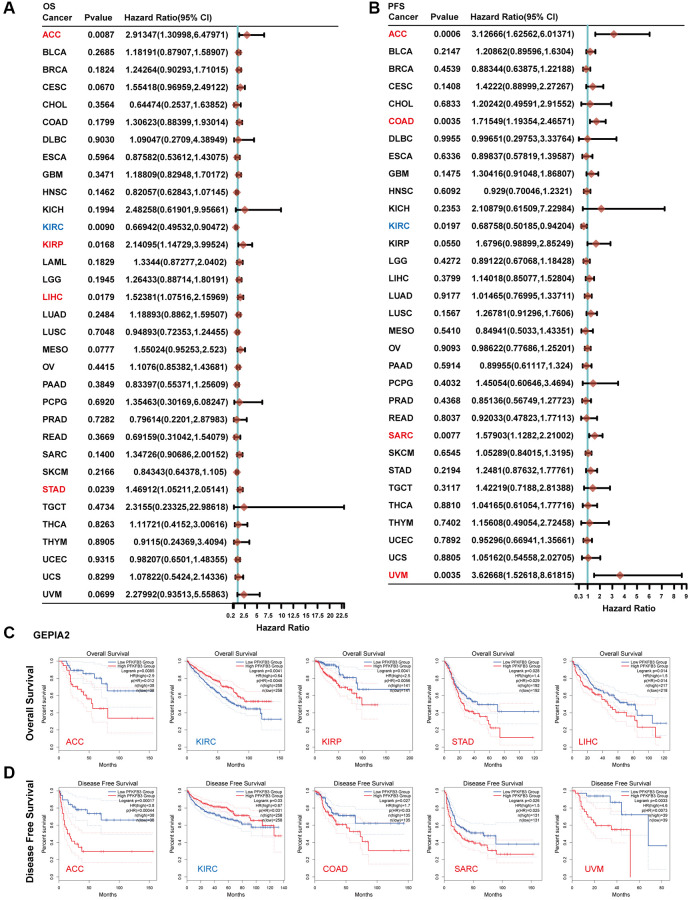
**The correlation between *PFKFB3* gene expression and survival prognosis in pan-cancer.** (**A**) Analyzing overall survival (OS) of various tumors in TCGA according to *PFKFB3* gene expression. (**B**) Analyzing Progression-free survival (PFS) of various tumors in TCGA according to *PFKFB3* gene expression. (**C**) We utilized the GEPIA2 to analyze OS of various tumors in TCGA according to *PFKFB3* gene expression. (**D**) We utilized the GEPIA2 to analyze disease-free survival (DFS) of various tumors in TCGA according to *PFKFB3* gene expression.

We explored the survival prognosis value of PFKFB3 using Kaplan-Meier, the overall survival (OS) results show that high expression of *PFKFB3* was significantly correlated to the poor OS for patients with adrenocortical carcinoma (ACC) (HR = 2.91, logrank *p* = 0.0087), KIRP (HR = 2.14, logrank *p* = 0.0168), STAD (HR = 1.47, logrank *p* = 0.0239), and LIHC (HR = 1.52, logrank *p* = 0.0179, Figure 2A), and the similar results were shown in Figure 2C. Progression-free survival (PFS) analysis showed high *PFKFB3* expression was significantly correlated with poor prognosis for TCGA cases of ACC (HR = 3.13, logrank *p* = 0.0006), COAD (HR = 1.72, logrank *p* = 0.0035), KIRP (HR = 1.68, logrank *p* = 0.0550), sarcoma (SARC, HR = 1.58, logrank *p* = 0.0077), and uveal melanoma (UVM, HR = 3.63, logrank *p* = 0.0035, Figure 2B), and disease-free survival (DFS) analysis showed similar results in Figure 2D. Moreover, low expression of the *PFKFB3* gene was significantly correlated with poor prognosis for KIRC (Figure 2).

### The characteristic of PFKFB3 mutation and the relationship between *PFKFB3* expression and TMB, MSI, and MMRs in pan-cancer

**Figure 3:**
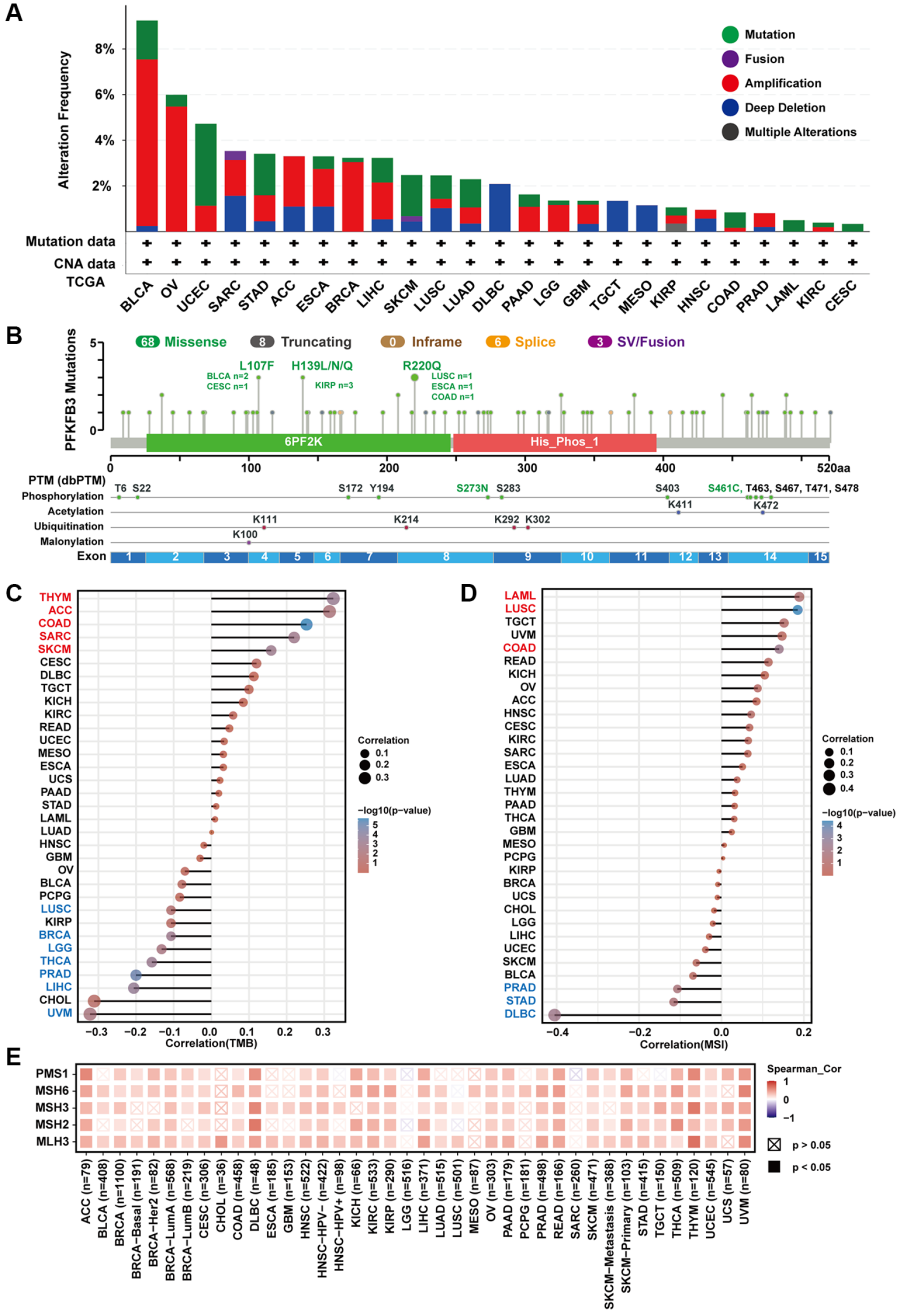
**The characteristic of PFKFB3 mutation and the relationship between TMB, MSI, and MMRs in pan-cancer.** (**A**) We utilized the cBioPortal tool to study the genomic alteration of *PFKFB3* for different tumors. (**B**) The characteristic of PFKFB3 mutations and posttranscriptional modification. (**C**) The relationship between *PFKFB3* expression and TMB in various malignancies. (**D**) The association between *PFKFB3* expression and MSI in pan-cancer. In Figure 3C and 3D, red fonts indicate a positive correlation and blue fonts indicate a negative correlation. (**E**) Correlation between *PFKFB3* expression and MMRs.

We investigated the genomic alteration and genetic modification of PFKFB3 in TCGA pan-cancer using the cBioPortal. The alteration frequency of *PFKFB3* was highest in BLCA tumors (<9%) and the second highest alteration frequency was observed in OV tumors (<6%), and their main type was “amplification” (Figure 3A). The type, site, and case numbers of *PFKFB3* genetic change and genetic modification were shown in Figure 3B. Furthermore, we have utilized TIMER 2.0 to analyze the correlation between mutated *PFKFB3* and immune infiltration in pan-cancer. The results showed that mutated *PFKFB3* was positively correlated with B cells and CD8+ T cells immune infiltration in UCEC (Supplementary Figure 5A), and positively correlated with macrophage immune infiltration in STAD (Supplementary Figure 5B). The mutated *PFKFB3* was negatively correlated with macrophage immune infiltration in LUSC (Supplementary Figure 5C).

TMB is a biomarker for immunotherapy, which could predict immune checkpoint inhibitors’ efficacy in numerous cancer types [12]. Our results show that *PFKFB3* expression was correlated positively with TMB in THYM, ACC, COAD, SARC, and skin cutaneous melanoma (SKCM); and correlated negatively with TMB in LUSC, BRCA, brain lower grade glioma (LGG), THCA, PRAD, LIHC, and UVM (Figure 3C). Furthermore, MSI acts as a predictor of response to immunotherapy and chemotherapy and is directly linked to tumor development [13]. Further analysis of *PFKFB3* expression indicated positive correlations with MSI in acute myeloid leukemia (LAML), LUSC, and COAD; and negative correlations with MSI in PRAD, STAD, and DLBC (Figure 3D).

MMR could maintain genome stability against spontaneous DNA damage [13]. MSI is caused by deficiencies in MMR. Furthermore, we study the correlation between *PFKFB3* expression and MMR. The results show that *PFKFB3* expression was positively correlated with five MMR genes, including PMS1 homolog 1, mismatch repair system component (PMS1), mutS homolog 6 (MSH6), mutS homolog 3 (MSH3), mutS homolog 2 (MSH2), and mutL homolog 3 (MLH3) (Figure 3E).

### The correlation between *PFKFB3* gene expression and immune infiltration in cancer

**Figure 4:**
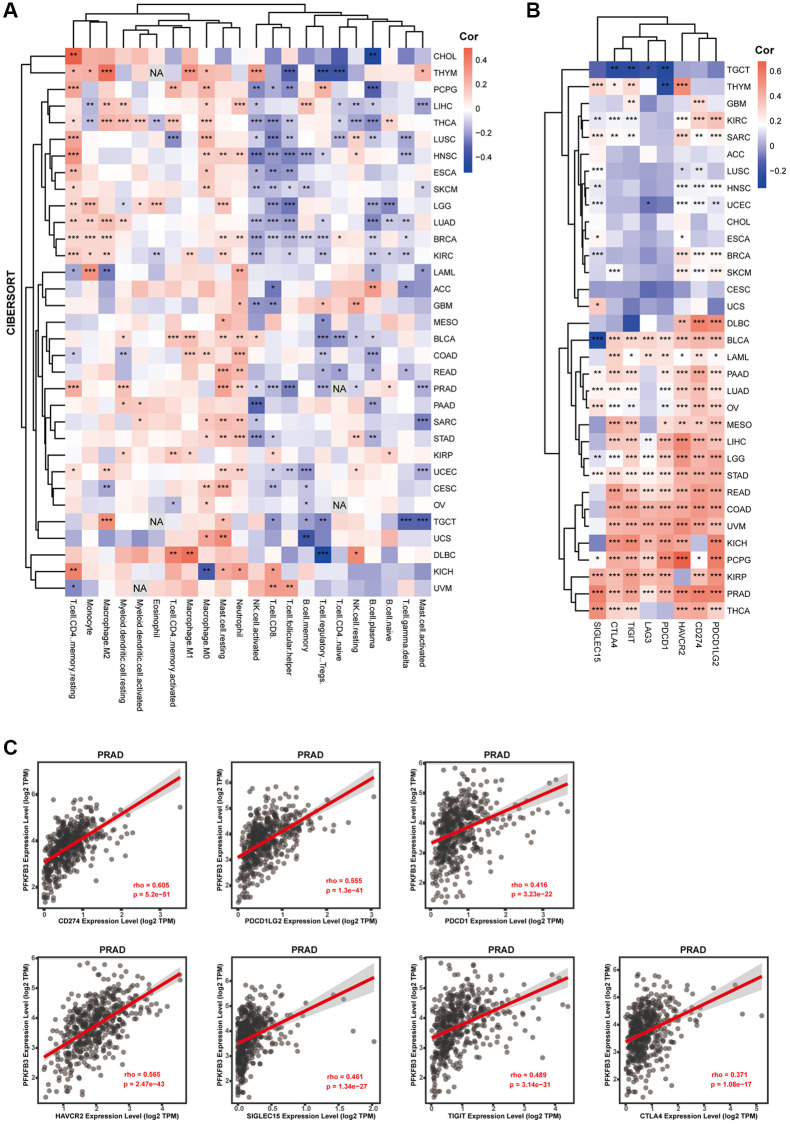
**The correlation between *PFKFB3* expression and immune infiltration or immune checkpoint in pan-cancer.** (**A**) Correlation analysis between *PFKFB3* expression and immunological infiltration in pan-cancer by CIBERSORT algorithm. (**B**) Correlation analysis between *PFKFB3* expression and immune checkpoint in pan-cancer. (**C**) The association between *PFKFB3* expression and CD274, PDCD1LG2, and PDCD1, HAVCR2, SIGLEC15, TIGIT, and CTLA4 in PRAD. All data ^*^P < 0.05; ^**^P < 0.01; ^***^P < 0.001.

TME is directly associated with treatment response and clinical prognosis of tumors [10]. Immune infiltration cells are regarded as one of the dominant elements of TME [14]. To explore immune cell infiltration of *PFKFB3* in cancers, we analyzed the correlation between the immune cell infiltration and *PFKFB3* expression in TCGA pan-cancer. Combining CIBERSORT and TIMER analysis, we found a significant positive association between *PFKFB3* expression level and neutrophil, macrophage, and myeloid dendritic cells infiltration in pan-cancer (Figure 4A and Supplementary Figure 6), and a negative correlation between *PFKFB3* expression level and NK cells and B cells infiltration in pan-cancer (Figure 4A and Supplementary Figure 6).

Immune checkpoint contributes to the evasion of the immune system by tumor cells. Therefore, immune checkpoint blockade therapy has become one of the major strategies in fighting cancer [15]. Next, we investigated the correlation between *PFKFB3* expression and the expression of immune checkpoint markers in pan-cancer, including programmed cell death 1 (PDCD1), programmed cell death 1 ligand 2 (PDCD1LG2), CD274, cytotoxic T-lymphocyte associated protein 4 (CTLA4), hepatitis A virus cellular receptor 2 (HAVCR2), lymphocyte activating 3 (LAG3), and sialic acid binding Ig like lectin 15 (SIGLEC15). Intriguingly, our results show that *PFKFB3* expression was remarkably significantly correlated with the expression of almost all these immune checkpoint markers in 17 types of tumors, including BLCA, LAML, PAAD, LUAD, OV, MESO, LIHC, LGG, STAD, READ, COAD, UVM, KICH, pheochromocytoma and paraganglioma (PCPG), KIRP, PRAD, and THCA (Figure 4B). *PFKFB3* expression correlated with CD274 (rho = 0.605, *p* = 5.2e-51), PDCD1LG2 (rho = 0.555, *p* = 1.3e-41), PDCD1 (rho = 0.416, *p* = 3.23e-22), HAVCR2 (rho = 0.565, *p* = 2.47e-43), SIGLEC15 (rho = 0.461, *p* = 1.34e-27), TIGIT (rho = 0.489, *p* = 3.14e-31), CTLA4 (rho = 0.371, *p* = 1.08e-17) expression in PRAD as a representative, respectively (Figure 4C).

### Analysis of the relationship of *PFKFB3* expression and immunoregulators in pan-cancer

**Figure 5:**
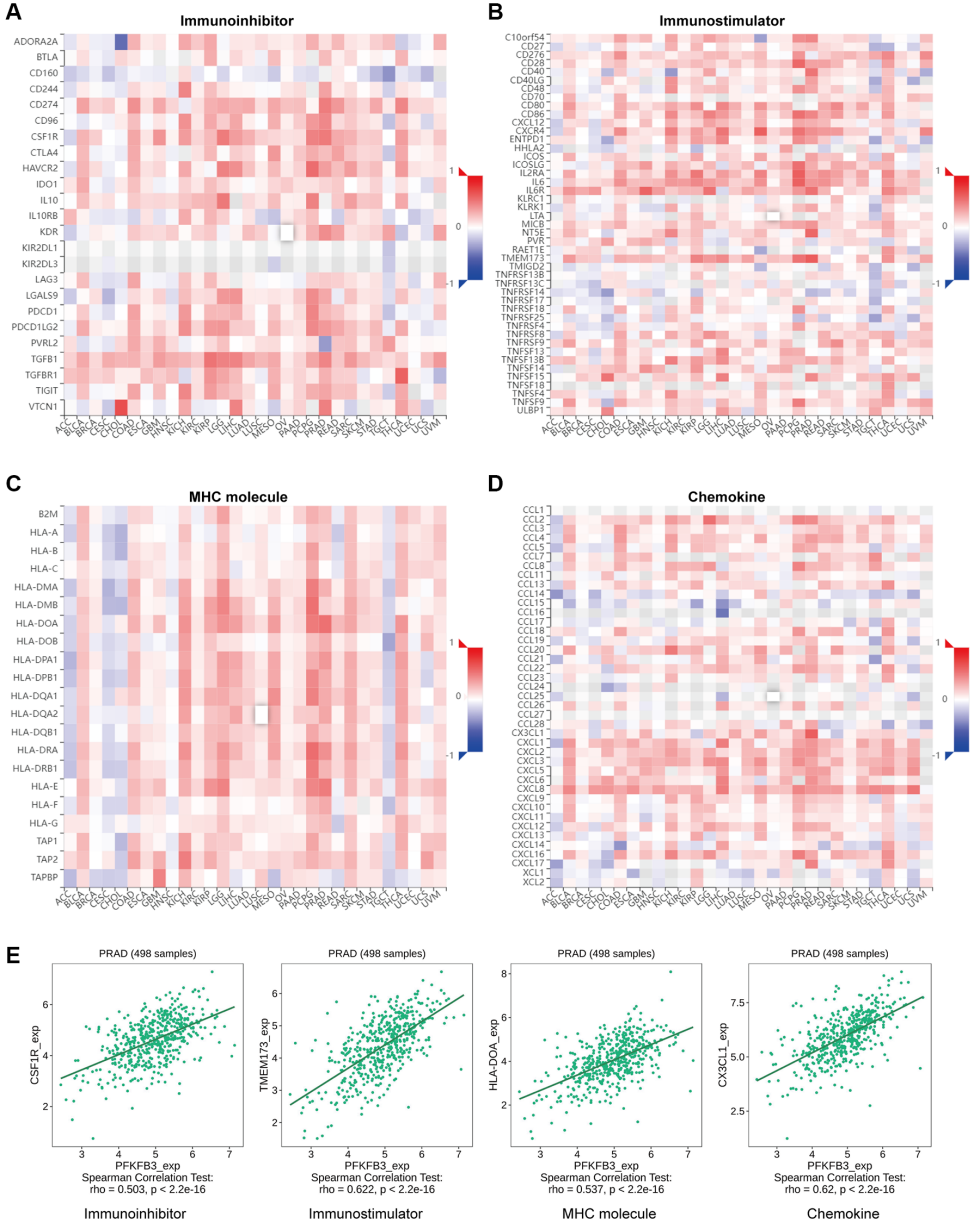
**The association between *PFKFB3* gene mutation and immune regulators in pan-cancer.** The heatmaps about the relationship between *PFKFB3* expression and immune markers: (**A**) immunoinhibitory factors; (**B**) immunostimulatory factors; (**C**) MHC molecule; (**D**) Chemokine. (**E**) The expression of *PFKFB3* correlated with corresponding immune markers (CSF1R, TMEM173, HLA-DOA, and CX3CL1) in PRAD.

Gene co-expression analysis was performed to study the relationship between *PFKFB3* and immunoregulators. The immune-related genes of immunoinhibitory factors (Figure 5A), immunostimulatory factors (Figure 5B), MHC molecule (Figure 5C), and chemokine (Figure 5D) were examined, respectively. Our results indicated that almost all the immunoregulators were significantly positively correlated with *PFKFB3* expression. The scatter plot shows that *PFKFB3* positively correlated with colony-stimulating factor 1 receptor (CSF1R) (rho = 0.503, *p* < 2.2e-16), transmembrane protein 173 (TMEM173) (rho = 0.622, *p* < 2.2e-16), major histocompatibility complex, class II, DO alpha (HLA-DOA) (rho = 0.537, *p* < 2.2e-16), and C-X3-C motif chemokine ligand 1 (CX3CL1) (rho = 0.62, *p* < 2.2e-16) expression in PRAD as a representative, respectively (Figure 5E).

### Single-cell analyzing the characteristic of the expression of *PFKFB3* in TME in pan-cancer

**Figure 6:**
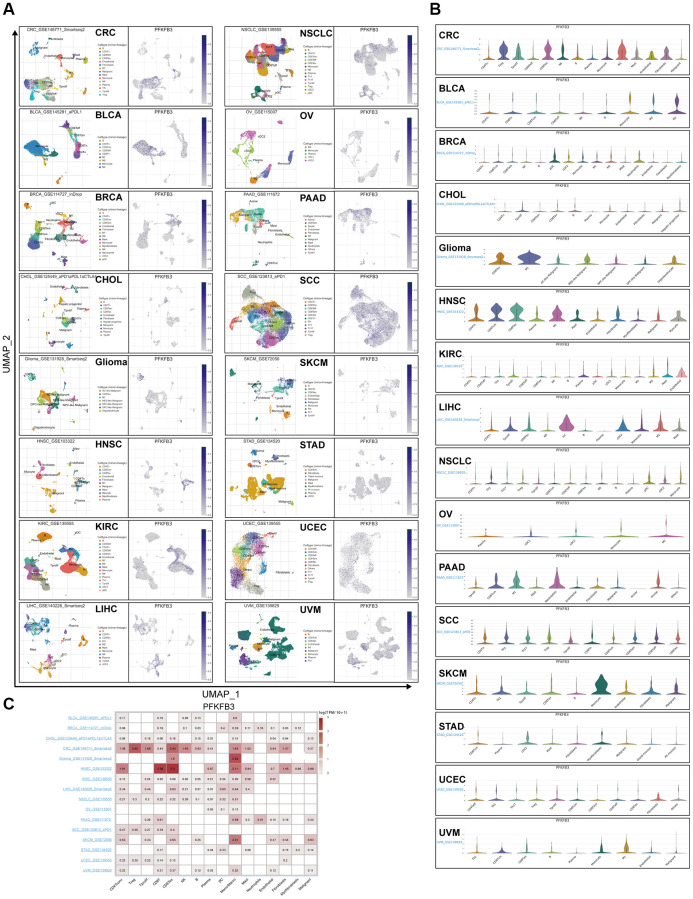
**Single-cell analyze the characteristic of the *PFKFB3* expression in pan-cancer.** (**A**) The visualized single-cell UMAP plots are to show the cell distribution of treatment response groups (left) and the expression of *PFKFB3* (right). (**B**) The grid violin plot reflects the distribution of *PFKFB3* expression in different cell types across all datasets with various cancers. (**C**) The heatmap reflects the distribution of *PFKFB3* expression in different cell types across all datasets with various cancers.

Tumor Immune Single Cell Hub (TISCH) is a large-scale curated database that integrates single-cell transcriptomic profiles of nearly 2 million cells from 76 high-quality tumor datasets across 27 cancer types, which contribute to the comprehensive exploration of TME [16]. Firstly, we utilize TISCH to visualize UMAP plots and explore the character of the expression of *PFKFB3* at the single-cell resolution in pan-caner (Figure 6A). Besides, we further analyzed the distribution of the *PFKFB3* expression in different TME cells in pan-cancer (Figure 6B). Finally, we explore the *PFKFB3* expression at the cell-type averaged level and display using a heatmap (Figure 6C). For cancer type, combining these results indicated that *PFKFB3* has a remarkably high expression in TME in colorectal cancer (CRC) and HNSC (Figure 6). For the TME cell type, these results indicated that *PFKFB3* has a remarkably high expression in Monocyte/Macrophage (Figure 6).

### Genes enrichment analysis of PFKFB3 in pan-cancer

**Figure 7:**
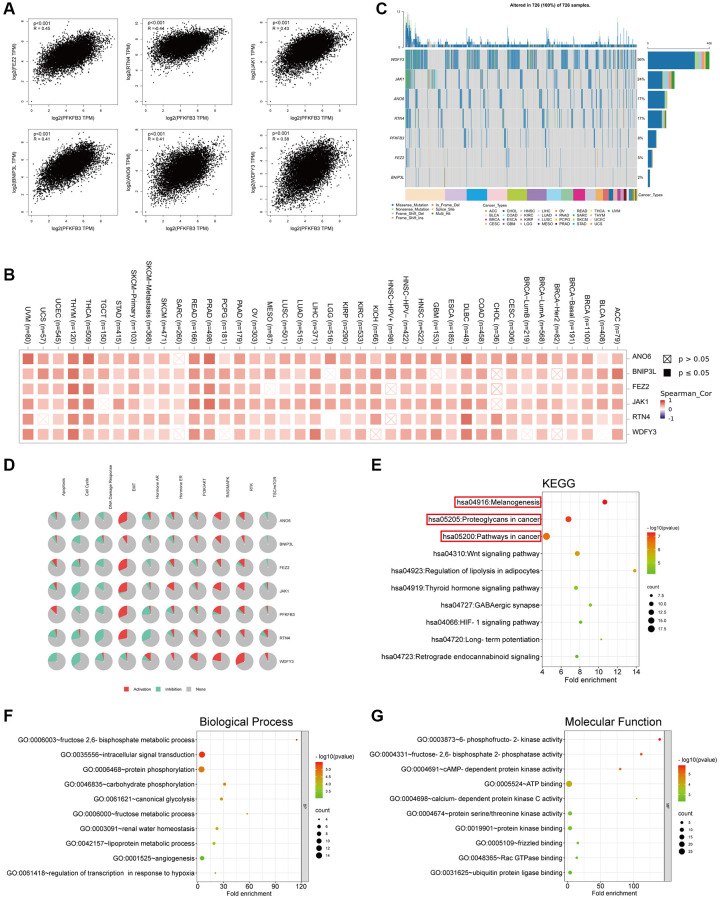
**Genes enrichment analysis of PFKFB3 in pan-cancer.** (**A**) The association between PFKFB3 and representative genes among the top *PFKFB3*-related genes analyzed by GEPIA2 in pan-cancer. (**B**) Heatmap shows the correlation between PFKFB3 and selected genes in pan-cancer. (**C**) Single Nucleotide Variation (SNV) frequency analysis of selected genes in pan-cancer. (**D**) Pathway activity analysis of selected genes in pan-cancer. (**E**) KEGG pathway analysis of PFKFB3-binding proteins and *PFKFB3*-correlated gene. (**F**, **G**) Go analysis of PFKFB3-binding proteins and PFKFB3-correlated gene, biological process (**F**), molecular function (**G**).

To analyze the roles of PFKFB3 in tumorigenesis, we screen out correlated genes of PFKFB3 in pan-cancer and the PFKFB3 interacting proteins for pathway enrichment analyses. And then, we used the GEPIA2 to obtain the top 100 *PFKFB3* correlated genes based on all types of cancers in the TCGA database. We found *PFKFB3* expression was positively correlated with Fasciculation and elongation protein zeta 2 (FEZ2, R-0.45), Reticulon 4 (RTN4, *R* = 0.44), Janus kinase-1 (JAK1, *R* = 0.43), Anoctamin-6 (ANO6, *R* = 0.41), BCL2/adenovirus E1B interacting protein 3-like (BNIP3L, *R* = 0.41), WD repeat and FYVE domain containing 3 (WDFY3) (*R* = 0.38, all *p* < 0.001, Figure 7A). The heatmap showed that *PFKFB3* expression was positively correlated with the above six genes almost in complete cancer types (Figure 7B). We used the GSCALite websites to analyze the function of these top 6 *PFKFB3* correlated genes in SNV frequency and pathway activity. SNV frequency data show the mutate frequency of WDFY3 (56%), JAK1 (24%), ANO6 (17%), RTN4 (17%), FEZ2 (5%), and BNIP3L (2%) (Figure 7C). Pathway activity analysis shows that these top 6 genes mainly activate epithelial-mesenchymal transition (EMT), RAS/MAPK, and Ras oncogene at 85D (RTK) pathways, and inhibit cell cycle and DNA damage response in pan-cancer (Figure 7D). Furthermore, we utilized the GSCALite and the cancer therapeutics response portal (CTRP) database to analyze the drug sensitivity of *PFKFB3*-related genes in KIRP (Supplementary Figure 7). The results showed that the expression of *PFKFB3* was highly correlated with drug sensitivity, which suggests that PFKFB3 has a potential to be a therapy target in tumors.

We utilized STRING to obtain the top 50 PFKFB3-binding proteins, which were supported by experimental evidence (Supplementary Figure 8) [17, 18]. We combined the two datasets, PFKFB3-binding proteins and top 100 PFKFB3 correlated genes, to analyze KEGG and GO enrichment. KEGG results showed that *PFKFB3* correlated genes were involved in melanogenesis, proteoglycans in cancer, and pathways in the cancer pathway (Figure 7E). The GO enrichment results suggested that *PFKFB3* correlated genes were linked to metabolic, intracellular signal transduction, and kinase activity (Figure 7F, 7G).

### Establishment and evaluation of prognostic risk models in KIRP

**Figure 8:**
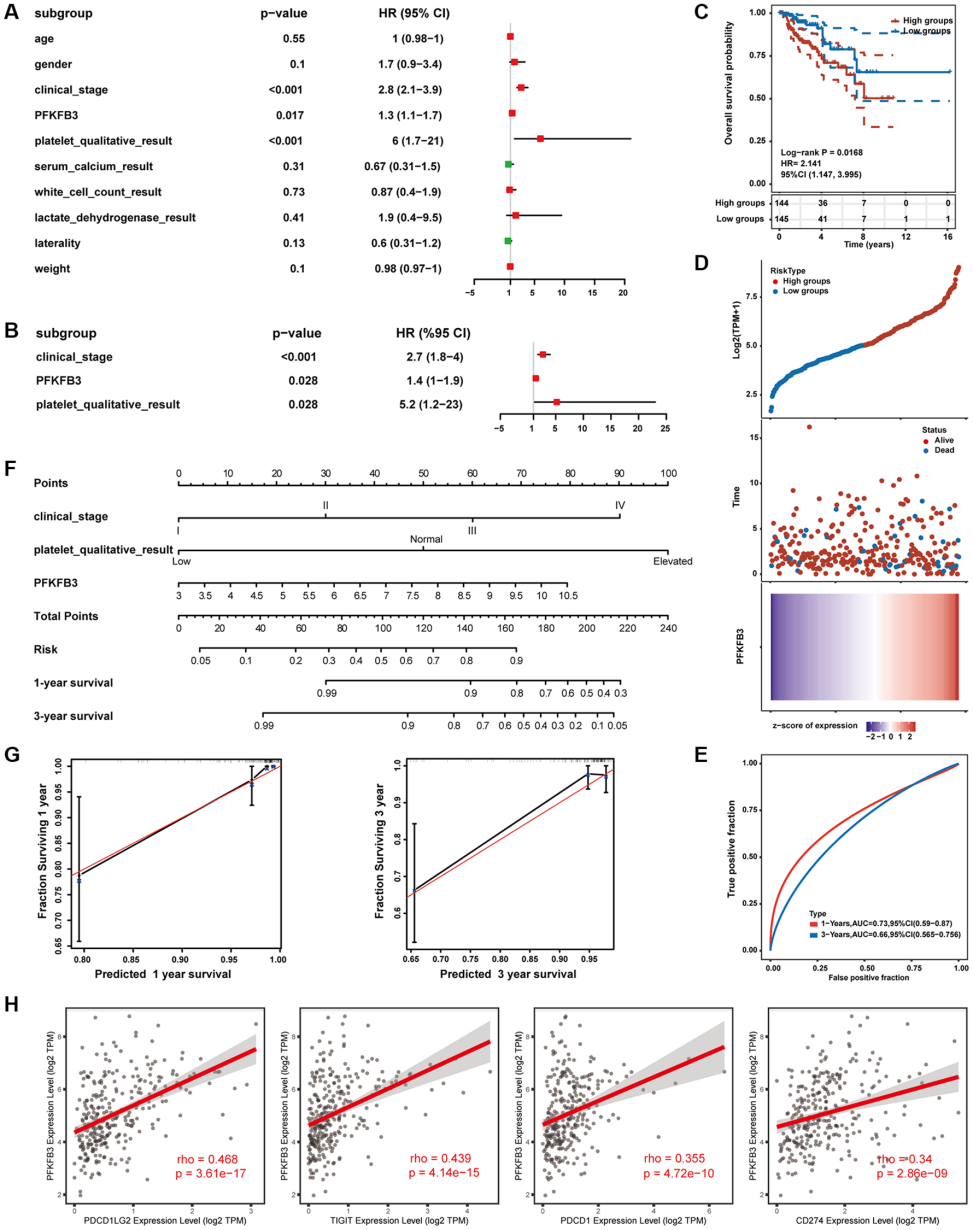
**Establishment and evaluation of prognostic risk models.** (**A**) Univariate Cox regression analysis. (**B**) Multivariate Cox regression analysis. (**C**) The survival status in high *PFKFB3* expression groups compared with low *PFKFB3* expression groups. (**D**) The distribution of risk score, the distribution of OS status, and the heatmap of PFKFB expression after risk score grouping. (**E**) Receiver operating characteristic (ROC) curve analysis. (**F**) The risk prognostic model was established based on prognostic factors of clinical_stage, platelet_qualitative_result, and expression level of *PFKFB3*. (**G**) Calibration plot for the risk prognostic model. (**H**) PFKFB3 was significantly positively correlated with immune checkpoints including PDCD1LG2, TIGIT, PDCD1, and CD274.

Furthermore, we collected KIRP expression data and clinical data from TCGA public databases. In univariate regression analysis, clinical_stage, platelet_qualitative_result, and expression level of *PFKFB3* were shown to be prognostic variables for the prognosis of OS in KIRP patients (Figure 8A). Moreover, multivariate regression Cox analysis indicated that *PFKFB3* expression was an independent prognostic factor for KIRP (Figure 8B). Therefore, we utilize the expression of the *PFKFB3* level to calculate the prognostic risk score. Then, the KIRP patients were divided into a high-risk group and a low-risk group by median of the risk score. The OS between different groups was compared by Kaplan-Meier analysis with the Log-rank test. The results suggested that the high-risk groups showed a poor prognosis compared with the low group (Figure 8C). The heatmap of prognosis signature after risk score grouping and the distribution of risk status and risk score were shown in Figure 8D. The 1-year and 3-year ROC curves were analyzed to evaluate the predictive accuracy of the PFKFB3 signature (Figure 8E). Moreover, the risk prognostic model was established based on prognostic factors of clinical_stage, platelet_qualitative_result, and expression level of *PFKFB3* (*P* < 0.05), and the 1-, 3-years survival was given (Figure 8F). The calibration curves of 1- and 3-year survival of risk indicated the model has a good predictive ability (Figure 8G). Finally, we found that PFKFB3 was involved in the regulation of the immune system process (Supplementary Figure 9), and PFKFB3 was significantly positively correlated with immune checkpoints including PDCD1LG2 (rho = 0.468, *p* = 3.61e-17), TIGIT (rho = 0.439, *p* = 4.14e-15), PDCD1 (rho = 0.355, *p* = 4.72e-10), and CD274 (rho = 0.34, *p* = 2.86e-09, Figure 8H) in KIRP. Combining these results implicated that PFKFB3 may play a key role in immune infiltration in the TME and as a valuable prognostic factor in KIRP.

## DISCUSSION

At present, it is still a big challenge to interpret the mechanism of tumor development and discover effective therapeutic strategies. TME is a key regulatory factor in tumors and contributes to the initiation, progression, and metastasis of tumors [10]. Prof. Warburg observed a phenomenon: normal cells rely primarily on mitochondrial oxidative phosphorylation to generate energy. However, most cancer cells instead rely on aerobic glycolysis, which is termed “the Warburg effect”, suggesting that glycolysis plays an important role in tumorigenesis [3]. PFKFB3 was a key regulatory enzyme in glycolysis. Multiple studies have indicated that PFKFB3 plays an important role in several malignancies. However, pan-cancer evidence has yet to be established to interpret the function of PFKFB3 in TME and the clinical prognosis of different cancers. Our study revealed novel insights into the function of PFKFB3 in tumorigenesis across thirty-three different tumors.

In this study, we conducted a comprehensive assessment of the relationship between *PFKFB3* expression, patient prognosis, and especially TME in cancers based on the TCGA database. Firstly, we explored the mRNA expression level, protein expression level, and protein phosphorylation of PFKFB3 in multiple tumors using TCGA, CPTAC, and GTEx databases. The expression of *PFKFB3* was significantly elevated in CHOL, COAD, HNSC, STAD, and THCA. In contrast, *PFKFB3* expression was significantly reduced in BLCA, BRCA, KICH, KIRC, KIRP, LIHC, LUAD, LUSC, PRAD, DLBC, and THYM. The phosphorylation of PFKFB3 (Ser22, Ser441, and Ser461) was significantly increased in most tumors. While the functional consequence of phosphorylation at Ser22 and Ser441 is not yet clear, Ser461 has been established as an important modification site on PFKFB3. Protein kinase AMP-activated catalytic subunit alpha 1 (AMPK) enhances the glycolytic activity of PFKFB3 by phosphorylating PFKFB3 at Ser461, and therefore, promoting the proliferation of cancer cells [19].

We explored the relationship between *PFKFB3* expression and the prognosis of different tumor patients. We found that high *PFKFB3* expression was linked to poor prognosis in patients with ACC, COAD, KIRP, LIHC, SARC, STAD, and UVM. Low expression of the *PFKFB3* gene was associated with poor prognosis for patients with KIRC. Aberrant expression of *PFKFB3* is frequently found in breast cancer, colon cancer, pancreatic cancer, gastric cancer, liver cancer, and many other neoplasms [11]. In many types of cancer, high expression of *PFKFB3* is associated with poor prognosis. PFKFB3 regulates tumor proliferation, invasiveness, and migration through different mechanisms. Previous studies reported that PFKFB3 impacts cancer cell proliferation by regulating the expression levels or post-transcriptional modification levels of cyclin-dependent kinase and thus influences cell cycle arrest in gastric cancer and cervical cancer [20–22]. *PFKFB3* knockdown inhibited hepatocellular carcinoma cell proliferation by impairing DNA repair functions [23]. Moreover, a recent study suggested that PFKFB3 may be a novel epithelial-mesenchymal transition inducer and regulates the invasion and migration in nasopharyngeal carcinoma progression [24].

The deficiency of MMR leads to ineffective protection from autogenetic DNA damage, which affects genome stability [13]. A deficiency of MMR results in high MSI. High MSI, as well as high TMB, leads to produce an increase in neoantigen, which could then be recognized by immune cells, and finally, improve immune responses [12]. Therefore, TMB, MSI, and MMR are treated as a biomarker to judge whether tumor patients are suitable for immunotherapy. Our results indicated that PFKFB3 was highly significantly correlated with TMB, MSI, and MMR in numerous tumors. These findings suggest that considering PFKFB3 expression when assessing suitability for immunotherapy may benefit patients with relevant cancers.

TME is a key regulatory factor in the tumor, which is composed of tumor cells and stromal cells, mainly including cancer-associated fibroblast cells, endothelial cells, and lymphocytes. TME contributes to a suitable growth environment for tumor and helps cancer cell immune escape, therefore, progressing initiation, progression, and metastasis of the tumor [10]. Lymphocyte infiltrating is a key component of TME. The infiltration of immune cells into tumors correlates with patient outcomes [25]. High infiltration of TIGIT+ CD8+ T cells indicated poor prognosis in muscle-invasive bladder cancer [26]. Lactate dehydrogenase A (LDHA) plays an important role in glycolysis and regulates the abundance of lactate. The high expression level of *LDHA* was correlated with CD8+ T cells, neutrophils, and dendritic cells infiltrating and showed poor survival in COAD patients [27]. A recent study showed that high expression of *PFKFB3* induces CD274 molecule (CD274) expression via activating the NF-κB signal pathway in monocytes, therefore inhibiting CD8+ T cell activity and poor prognosis in hepatocellular carcinoma patients [28]. PFKFB3-NF-κB signaling induced the production of CXCL2 and CXCL8 in tumor-infiltrating monocytes, increased levels of CXCL2 and CXCL8 in monocytes and promote infiltration of oncostatin M-producing neutrophils in human hepatocellular carcinoma tissues [29]. We found a significant positive association between *PFKFB3* expression level and neutrophil, macrophage, and myeloid dendritic cells infiltration in pan-cancer; and a negative correlation between *PFKFB3* expression level and NK cells and B cells infiltration in pan-cancer. Moreover, our results show that *PFKFB3* expression was remarkably positively correlated with the expression of all 7 immune checkpoint markers in 17 types of tumors, including BLCA, LAML, PAAD, LUAD, OV, MESO, LIHC, LGG, STAD, READ, COAD, UVM, KICH, PCPG, KIRP, PRAD, and THCA, which indicated that PFKFB3 has a potential function to progress immune escape. Therefore, *PFKFB3* expression has an opportunity to be treated as a therapeutic synergy target of an immune-checkpoint inhibitor. Furthermore, PFKFB3 positively correlated with immunoinhibitory factors, immunostimulatory factors, MHC molecule, and chemokine. Finally, single-cell analysis has shown the characteristic of the expression of *PFKFB3* on different types of immune cells of TME in pan-cancer. Summary, these results confirmed that PFKFB3 is a potent regulatory factor for the TME, as it could regulate interactions between immune cells and tumors. However, the specific molecular mechanism of the crosstalk of PFKFB3 and TME is still unclear, and further research is needed to confirm.

We screen PFKFB3-binding proteins and *PFKFB3* correlated genes across all tumors for enrichment analyses. The KEGG pathway results identified that *PFKFB3* correlated genes were involved in melanogenesis, proteoglycans in cancer, and pathways in cancer. Pathway activity analysis showed that *PFKFB3* correlated top 6 genes mainly activate EMT, RAS/MAPK, and RTK pathway, and inhibit cell cycle and DNA damage response in pan-cancer. These pathways are reflected in previous research. EMT has been implicated in carcinogenesis and confers metastatic properties upon cancer cells by enhancing mobility, invasion, and resistance to apoptotic stimuli [30]. Overexpression of *PFKFB3* positively modulated cell proliferation, migration, and EMT in GC cells by activation of NF-κB signaling [31]. Inhibition of RAS down-regulates HIF-1alpha and reduces *PFKFB3* expression and might therefore block invasiveness, survival, and angiogenesis in Glioblastoma multiforme [32]. PFKFB3 is a hub for coordinating cell cycle and glucose metabolism by binding CDK4 and inhibiting the degradation of CDK4 in breast cancer [33]. A key role for PFKFB3 enzymatic activity in homologous recombination repair was confirmed, a selective PFKFB3 inhibitor that could potentially be used as a strategy for the treatment of cancer [7]. The analysis of the pathway of PFKFB3 in pan-cancer can be used as a future reference for exploring clinical tumor therapy.

Finally, we collected KIRP expression data and clinical data from TCGA public databases. We confirmed that PFKFB3 was an independent prognostic factor for KIRP, and established a risk prognostic model based on the expression of *PFKFB3* and clinical risk factor, which has a good predictive ability. A recent study indicated that *PFKFB3* expression is an independent prognostic factor in HCC via multivariate analysis [23, 34]. Moreover, the significant correlation between the expression of *PFKFB3* and immune cell infiltration was examined in KIRP. Notably, PFKFB3 was significantly positively correlated with immune checkpoints including PDCD1LG2, TIGIT, PDCD1, and CD274. These results indicated that PFKFB3 might interact with immune checkpoint and immune cell infiltration inflecting TME, and therefore progress cancer cell escape and affect the patient prognosis.

## CONCLUSIONS

In this study, we conducted a comprehensive analysis of the relationship between *PFKFB3* expression, patient prognostic, TMB, MSI, MMR, and especially TME in pan-cancer base on TCGA and GEO databases. We evidence the predictive ability of PFKFB3 in the prognosis of KIRP. Our study suggested that PFKFB3 is a potent regulatory factor for the TME and has the potential to be a valuable prognostic biomarker in human tumor therapy. This study’s majority of conclusions were based on the bioinformatic assay, which has some limitations. Therefore, further experimental studies are required to validate these conclusions to evidence the function of PFKFB3 in various tumors.

## MATERIALS AND METHODS

### Gene expression analysis

We used TIMER2.0 (http://timer.cistrome.org/) [18, 35–38] to analyze the expression level of *PFKFB3* between tumor and non-tumor tissues in different TCGA cancers. For tumors without non-tumor or with limited numbers of non-tumor tissues in TCGA, we used GEPIA2 (http://gepia2.cancer-pku.cn/#analysis) [39] to analyze the expression difference of *PFKFB3* between tumor tissues and the non-tumor tissues, under the settings of log2 (fold change) cutoff = 1, *P*-value cutoff = 0.01, and “Match TCGA normal and GTEx data.”

We analyze *PFKFB3* expression of different pathological stages in TCGA tumors via GEPIA2. The log2 [TPM (transcripts per million) + 1] transformed expression data were applied for the box or violin plots [40].

### Protein expression and phosphorylation analysis

The UALCAN (http://ualcan.path.uab.edu/index.html) tool can analyze cancer Omics data. We used UALCAN and the Clinical Proteomic Tumor Analysis Consortium (CPTAC) dataset to conduct protein expression analysis [18, 41]. We examined the expression level of total PFKFB3 protein or the phosphorylated proteins (phosphorylation at S22, S461, and S441, NP_001300992.1) between primary tumor and non-tumor tissues. Seven tumor datasets can be used in this web, including breast cancer, ovarian cancer, colon cancer, clear cell renal cell carcinoma, UCEC, lung adenocarcinoma (LUAD), and pediatric brain cancer, respectively [18].

### Survival analysis

We used the “Survival Analysis” module of GEPIA and Assistant for Clinical Bioinformatics (ACB, https://www.aclbi.com/) to analyze the overall survival (OS), progression-free survival (PFS), and disease-free survival (DFS) on *PFKFB3* expression in different tumors. We used a cut-off value of 50% to classify the high-expression and low-expression cohorts. The log-rank test was used in the hypothesis test [18].

### Genetic changes, SNV frequency, and drug sensitivity analysis

We used the cBioPortal database (https://www.cbioportal.org/) to queries of the genetic alteration characteristics of *PFKFB3* [18, 42, 43] and chose the “TCGA Pan-Cancer Atlas Studies,” composed of 32 studies including 10967 samples. We used the “Cancer Types Summary” module of the cBioPortal database to analyze the alteration frequency, mutation type, and copy number alteration (CNA) across all TCGA tumors [18].

We used the GSCALite (http://bioinfo.life.hust.edu.cn/web/GSCALite/) to analyze SNV frequency, pathway activity, and drug Sensitivity [44].

### TMB and MSI analysis

TMB and MSI scores were calculated based on mutational information from TCGA. We explored the correlation between *PFKFB3* expression and TMB as well as MSI using Spearman’s method.

### Immune infiltration analysis

We utilized TIMER and CIBERSORT methods to analyze the correlation of *PFKFB3* expression and immune infiltration level in all TCGA tumors. We focused on 22 types of immune cells and cancer-associated fibroblast cells using Spearman’s Rho method. The *P-*values and Rho values were obtained via the purity-adjusted Spearman’s Rho. *P* < 0.05 was the significance threshold. The data were visualized as a scatter plot or a heatmap [18].

### The correlation analysis of immunoregulators in pan-cancer

We used the TISIDB (http://cis.hku.hk/TISIDB/index.php) to explore the correlation of *PFKFB3* with immune regulators, MHC molecules, and chemokine [45].

### Single-cell analysis

Tumor Immune Single Cell Hub (TISCH) is a large-scale curated database that integrates single-cell transcriptomic profiles of nearly 2 million cells from 76 high-quality tumor datasets across 27 cancer types, which contribute to the comprehensive exploration of TME [16]. We utilized TISCH to study the characteristic of the expression of *PFKFB3* in TME in pan-cancer.

### *PFKFB3*-related gene enrichment analysis

We utilized STRING (https://string-db.org/) to obtain 50 proteins, which interacted with PFKFB3 [17, 18]. And then, we used the “Similar Gene Detection” module of GEPIA2 to obtain the top 100 *PFKFB3*-correlated targeting genes based on pan-cancer in the TCGA database [18]. Next, we used GEPIA2 to analyze the correlation assay and used TIME2.0 to supply the heatmap data of the *PFKFB3*-correlated gene [18]. Finally, to analyze the function and pathway of the *PFKFB3*-correlated gene, we performed Gene Ontology (GO) analyses on DAVID (https://david.ncifcrf.gov/tools.jsp) [18]. We utilized Metascape (http://www.metascape.org/) to analyze the *PFKFB3* co-expression immune genes network of enrichment.

### Prognostic risk model modeling and evaluation

In this study, we collected kidney renal papillary cell carcinoma (KIRP) expression data and clinical data from TCGA (https://portal.gdc.cancer.gov/) public databases. Age, gender, clinical stage, platelet, serum calcium, white cell count, lactate dehydrogenase, laterality, weight, and the expression level of *PFKFB3* were included in the univariate Cox regression analysis. And statistically significant (*p* < 0.05) was selected as prognostic factors to perform multivariate Cox regression analysis. And then the establishment of the prognostic risk model utilizes the above prognostic factors, provides the risk score, and plots the nomogram. The “survivalROC” R package was used to perform the time-dependent receiver operating characteristic curves (ROC) [46].

### Data availability statement

The data can be found in TIMER2.0 (http://timer.cistrome.org/), GEPIA2 (http://gepia2.cancer-pku.cn/#analysis), UALCAN (http://ualcan.path.uab.edu/index.html), cBioPortal (https://www.cbioportal.org/), GEPIA2021 (http://gepia2021.cancer-pku.cn/sub-expression.html), Jvenn (http://www.bioinformatics.com.cn), STRING (https://string-db.org/), DAVID (https://david.ncifcrf.gov/tools.jsp), TISIDB (http://cis.hku.hk/TISIDB/index.php), TISCH (http://tisch.comp-genomics.org/home/), Metascape (http://www.metascape.org/), GSCALite (http://bioinfo.life.hust.edu.cn/web/GSCALite/), and ACB (https://www.aclbi.com/). Further inquiries can be e-mailed to the corresponding authors.

## Supplementary Materials

Supplementary Figures
