# Supplementary material for: Glycolytic regulatory enzyme PFKFB3 as a prognostic and tumor microenvironment biomarker in human cancers
Source: Aging (Albany NY). 2023 May 30;15(10):4533–59. doi: 10.18632/aging.204758 (PMC10258027; doi:10.18632/aging.204758)
Supplement: Supplementary Figures [file aging-15-204758-s001.pdf]

SUPPLEMENTARY FIGURES

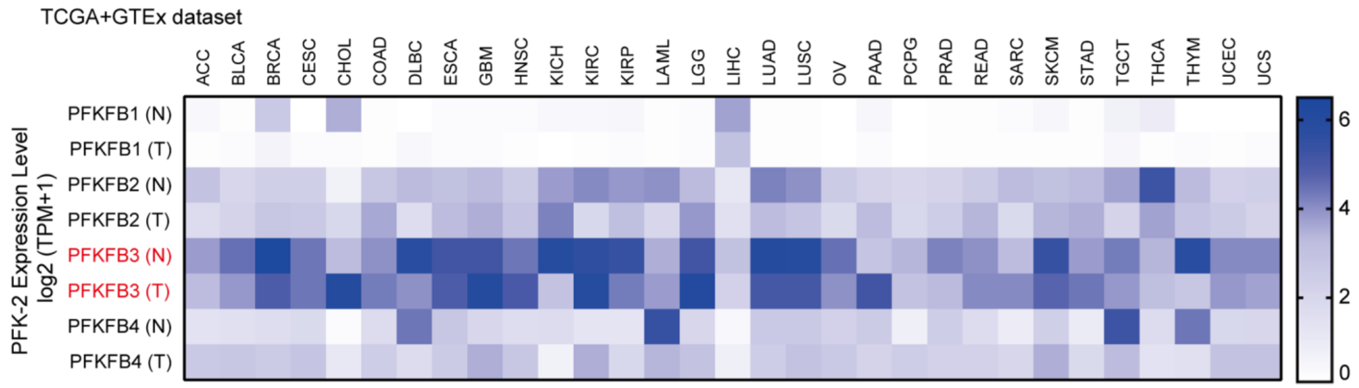

Supplementary Figure 1. The mRNA expression level of PFK-2 family genes. The heatmap of PFK-2 family genes, including *PFKFB1, PFKFB2, PFKFB3,* and *PFKFB4*, the expression level in tumor and non-tumor patients.

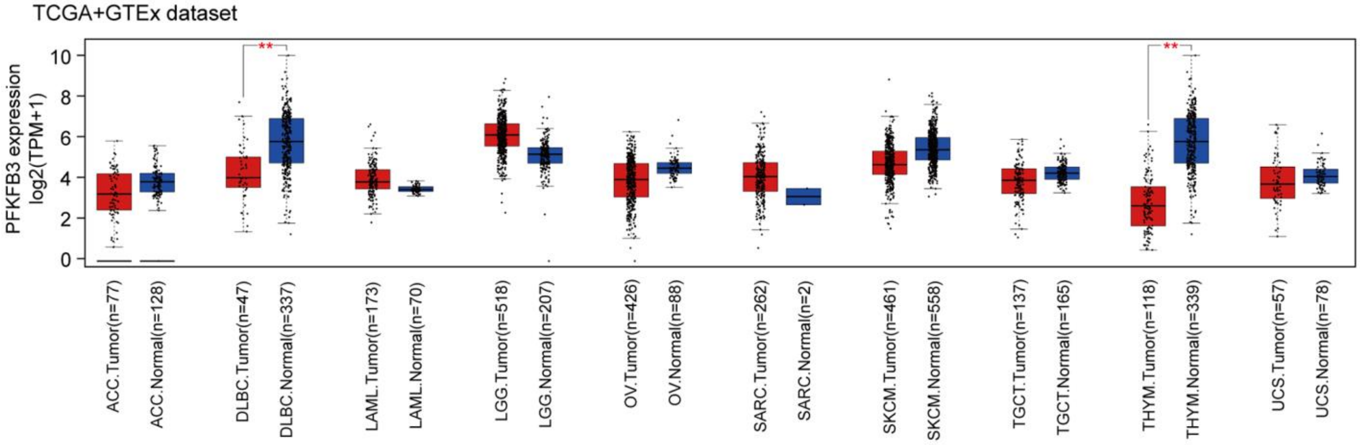

Supplementary Figure 2. The expression level of *PFKFB3* in pan-cancer. Combining TCGA and GTEx datasets, we further analyze the expression level of *PFKFB3* in ACC, DLBC, LAML, LGG, OV, SARC, SKCM, TGCT, THYM, and UCS.

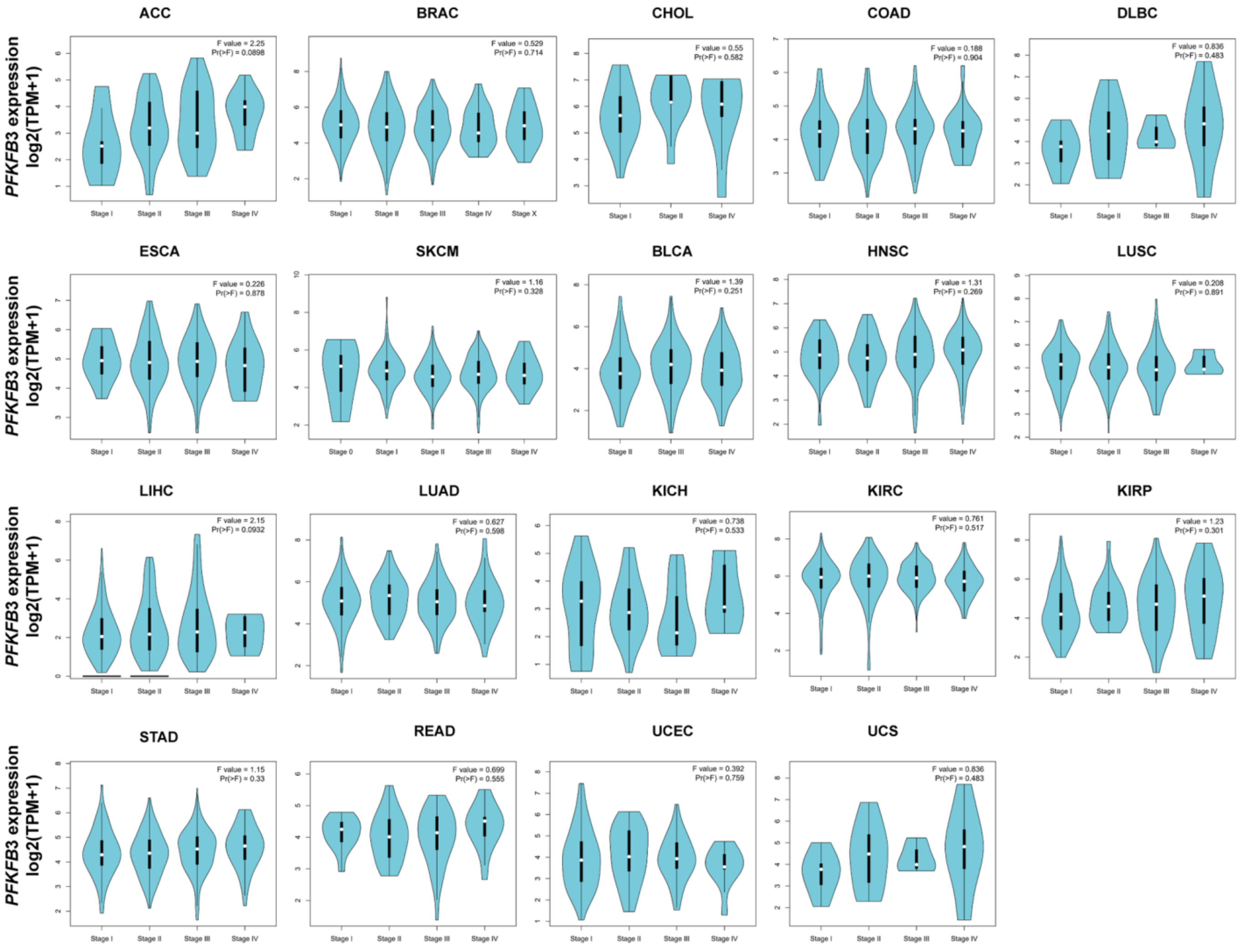

Supplementary Figure 3. *PFKFB3* gene expression in the pathological stage in pan-cancer. We utilized GEPIA2 to analyze the characteristic of *PFKFB3* expression in the pathological stage of pan-cancer.

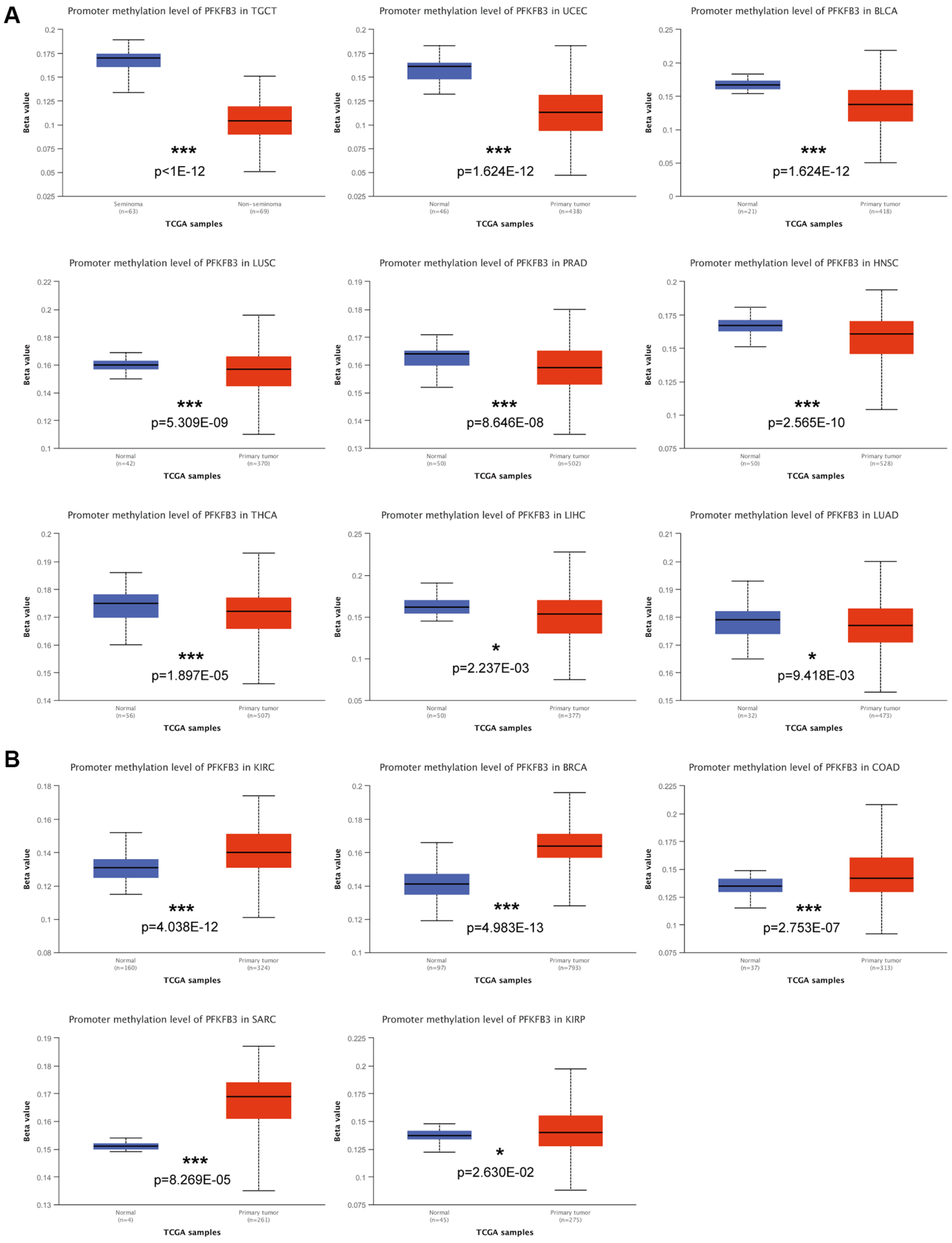

Supplementary Figure 4. The methylation levels of *PFKFB3* in pan-cancer. UALCAN database to investigate the promoter methylation level of *PFKFB3* in human pan-cancer. (**A**) The promoter methylation level of *PFKFB3* was significantly decreased. (**B**) The promoter methylation level of *PFKFB3* was significantly increased. $^{*}P < 0.05$; $^{**}P < 0.01$; $^{***}P < 0.001$.

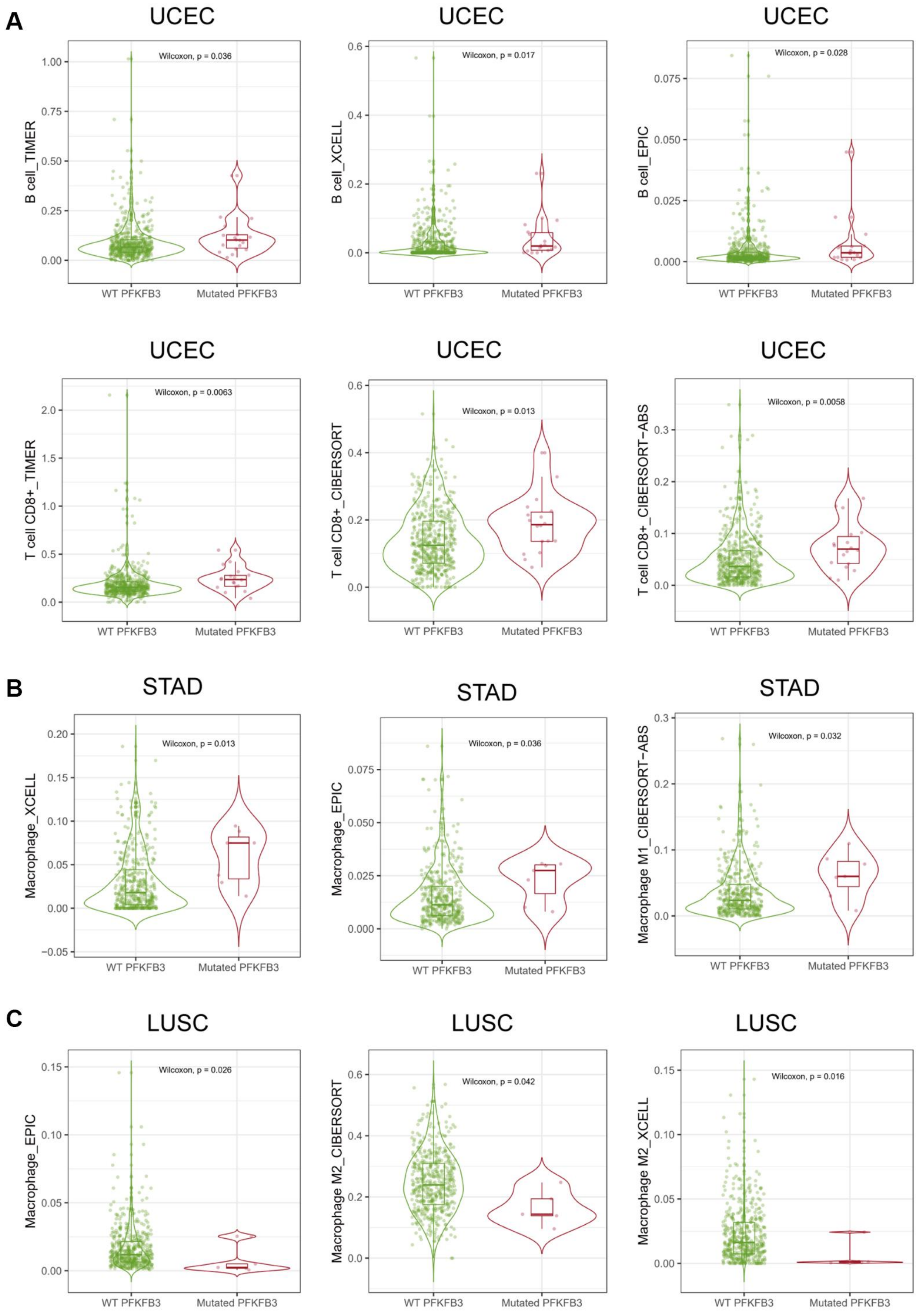

Supplementary Figure 5. The correlation of mutated PFKFB3 and the immune infiltration. We utilized TIMER 2.0 to analyze the correlation of mutated *PFKFB3* and the immune infiltration in UCEC (**A**), STAD (**B**), and LUSC (**C**).

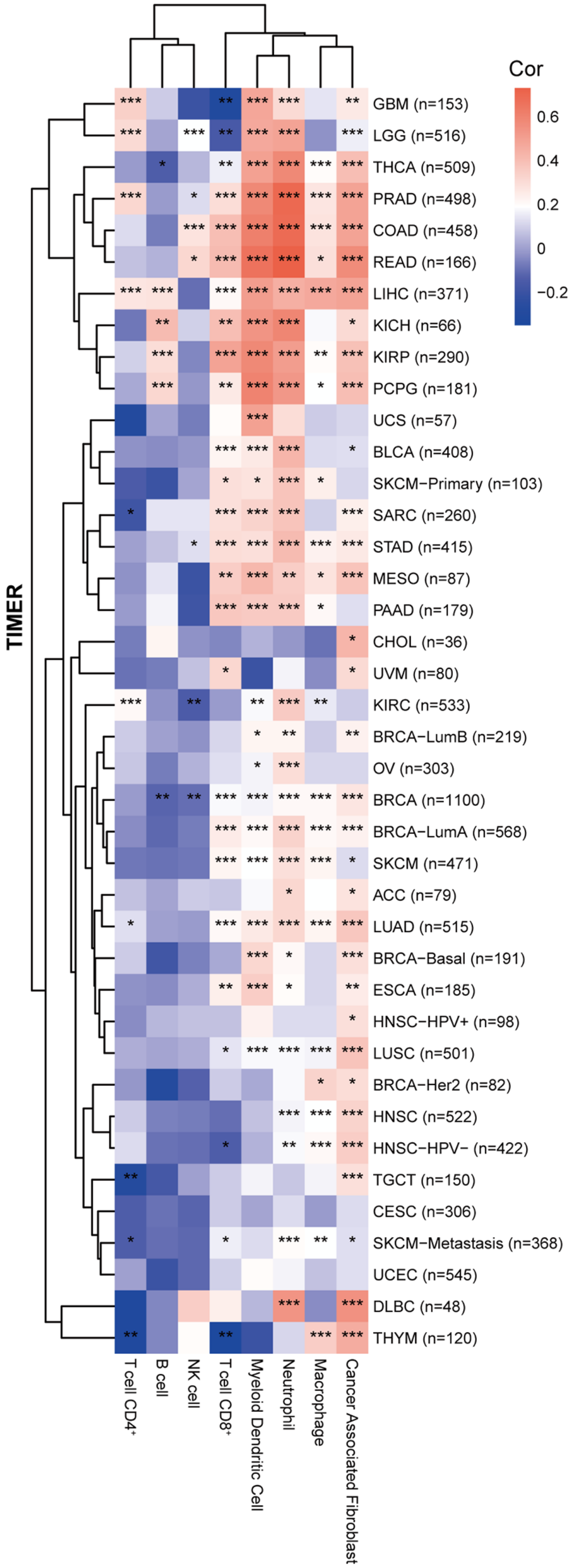

Supplementary Figure 6. The correlation between *PFKFB3* expression and immune infiltration. Correlation analysis between *PFKFB3* expression and immunological infiltration in pan-cancer by TIMER algorithm.

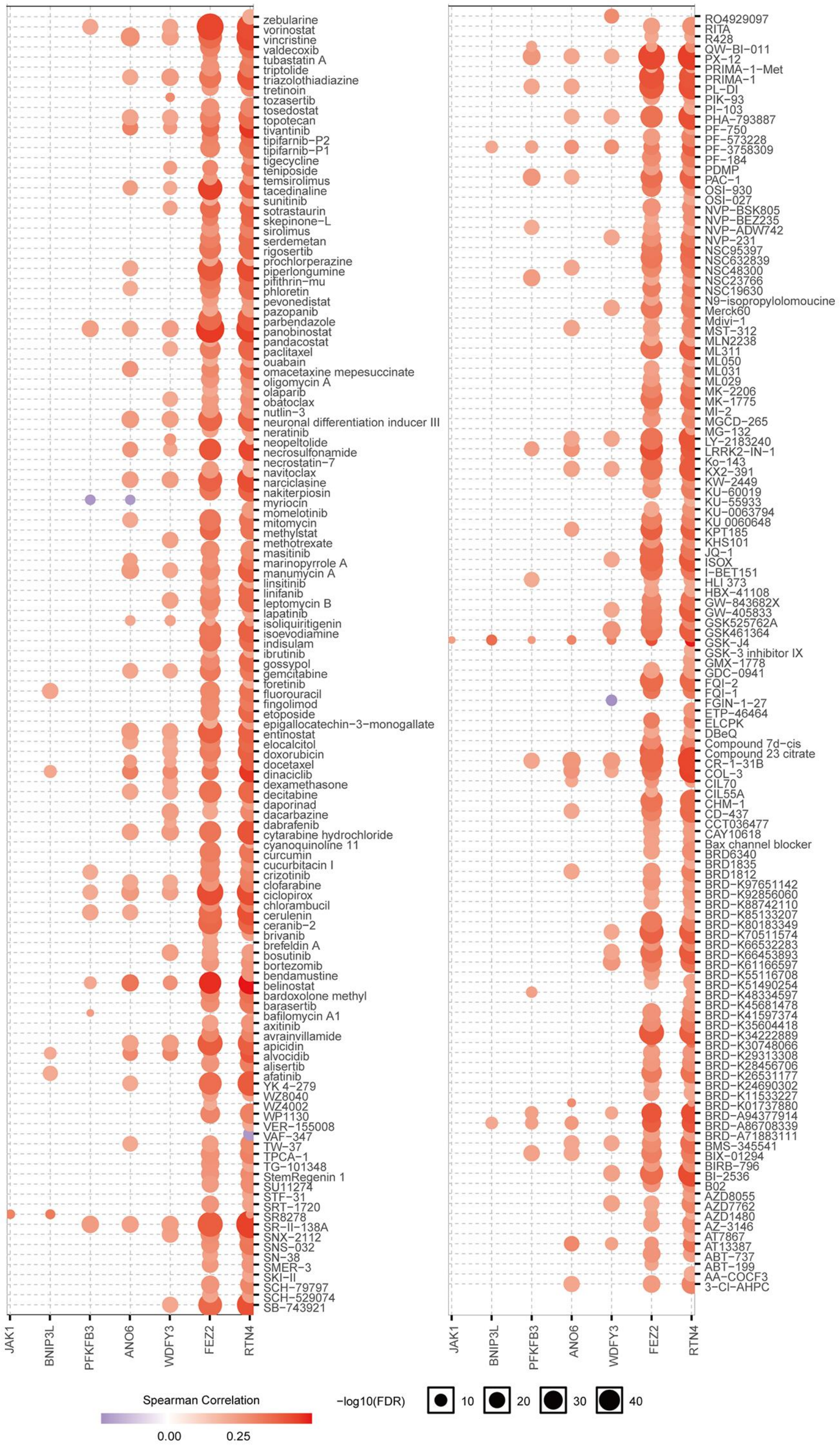

Supplementary Figure 7. Drug sensitivity analysis associated with *PFKFB3*-related genes in pan-cancer. We utilized the GSCALite and the CTRP database to analyze the drug sensitivity of *PFKFB3*-related genes in pan-cancer.

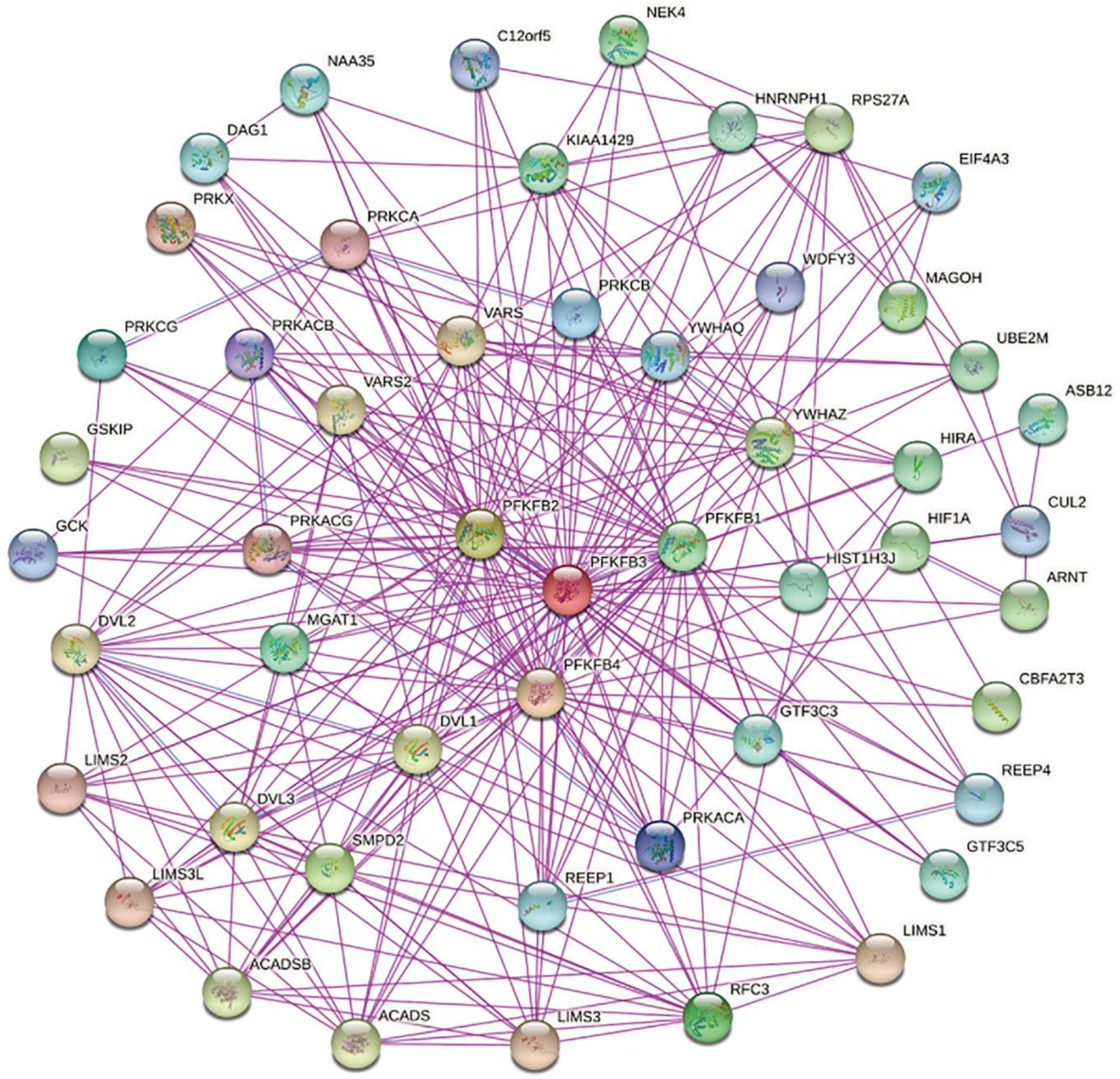

Supplementary Figure 8. The interaction network of the PFKFB3-binding proteins. We utilized STRING to obtain the interaction network of the top 50 *PFKFB3*-binding proteins.

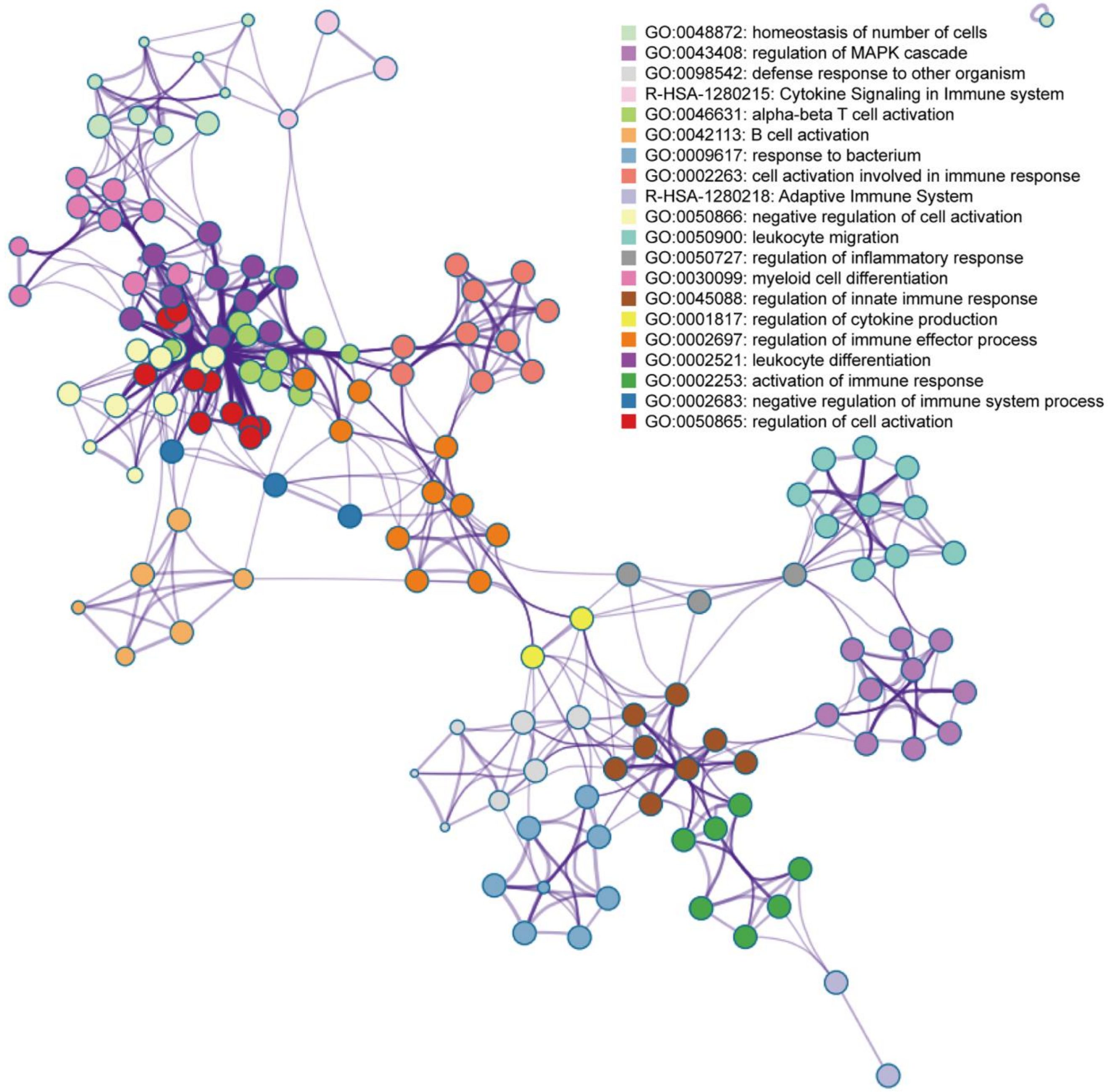

Supplementary Figure 9. PFKFB3 involved in the regulation of immune system process in KIRP. We utilized Metascape to analyze the *PFKFB3* co-expression immune genes network of enrichment. The network is colored by cluster ID.
